# Bifilm Inclusions in High Alloyed Cast Iron

**DOI:** 10.3390/ma14113067

**Published:** 2021-06-04

**Authors:** Marcin Stawarz, Malwina Dojka

**Affiliations:** Department of Foundry Engineering, Silesian University of Technology, 7 Towarowa Street, 44-100 Gliwice, Poland; malwina.dojka@polsl.pl

**Keywords:** bifilms, spheroidal graphite, alloyed cast iron, crystallization

## Abstract

Continuous improvement in the quality of castings is especially important since a cast without defects is a more competitive product due to its longer lifecycle and cheaper operation. Producing quality castings requires comprehensive knowledge of their production, crystallization process, and chemical composition. The crystallization of alloyed ductile iron (without the addition of magnesium) with oxide bifilm inclusions is discussed. These inclusions reduce the quality of the castings, but they are a catalyst for the growth of spheroidal graphite that crystallizes in their vicinity. The research was carried out for cast iron with a highly hyper-eutectic composition. Scanning electron microscopy and EDS analysis were used in the research. A detailed analysis of the chemical composition was also carried out based on the spectrometric method, weight method, etc. Based on the obtained results, a model of spheroidal graphite crystallization near bifilm inclusions was proposed. The surface of the analyzed graphite particles was smooth, which suggests a primary crystallization process. The phenomenon of simple graphite and bifilm segregation towards the heat center of the castings was also documented.

## 1. Introduction

**Figure 1:**
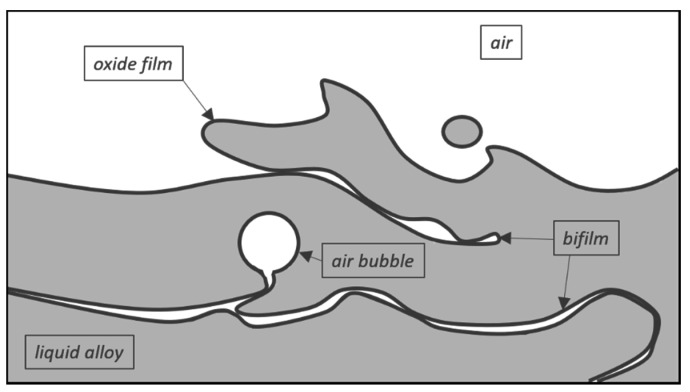
Scheme of bifilms and bubbles formation [8].

Foundry engineering processes are prone to many issues during casting manufacturing that may influence the final casting quality. Many are often ignored by manufacturers or even unknown. It is extremely important to realize the cause of poor casting quality before buying expensive additives for metal improvement and waste money on defective castings. In many works, researchers explain that the design of a proper gating system, the way that liquid metal is poured into a mold, and melt treatments are important factors that affect a casting’s quality [1,2,3,4,5,6]. Most of this research is based on Professor John Campbell’s bifilm theory [6,7,8]. In short, as one of the researchers explains [5], according to this theory, turbulence during the pouring generates defects inside the alloy as air bubbles and oxide films form on the surface of the liquid metal. The oxide film on the surface may fold over on itself. The double oxide ‘bifilm’ acts as a crack in the liquid metal, leading to the initiation of additional cracks and hot tears in the casting. Figure 1 presents a schematic illustration of surface turbulence causing the entrainment of bifilms and associated bubbles.

Dispinar D. et al. [1] and G. Gyarmati et al. [2] explained that the bifilm content in the melt is much more important than the dissolved gas content such as hydrogen, which has always been considered to be the major source of defects. They proved that with proper degassing, bifilm inclusions can be eliminated from the melt, and regardless of the hydrogen content, aluminium castings without defects can be manufactured. Similarly, Tiryakioğlu M. [4] concluded that if air entrainment defects are eliminated, there is no reason to measure or control the hydrogen levels in the melt. He clarified that due to the presence of bifilms with two unbonded interfaces, nucleation is bypassed during pore formation. Hydrogen diffuses to oxide bifilms and inflates them, therefore, hydrogen serves as an agent to make entrainment defects visible. What is even more interesting, Uludağ M. et al. [3] studied Al-7%Si-Mg alloy and discovered that the best melt quality was obtained when the melt was degassed without additives. Moreover, their research showed that all examined melt additions decreased the melt quality. They explained that Ti-containing intermetallic compounds were nucleated on bifilms. On the other hand, the addition of Sr to A356 increased the number of bifilms in the melt and also formed larger pores. Sr reacts with Al_2_O_3_, and the formation of complex compounds results in the fracturing of oxides into smaller pieces through breakaway oxidation.

Most research in this area has been conducted on nonferrous alloys, but bifilm defects are also a cause of problems with cast iron. Our previous work showed that bifilm inclusions may cause difficulties with the inoculation of high-chromium cast iron [9]. During inoculation with Ti addition, the TiC compounds that serve as underlays for M7C3 carbides are transported on the bifilms with the crystallization front to the shrinkage cavities. Thus, they are no longer an active nucleus in this location, which is very unfortunate from an economic viewpoint. According to previous investigations, bifilm defects are also present in the microstructure of grey cast iron. Professor J. Campbell wrote [10] that graphite may grow on bifilm inclusions. He explains that the silica film, when entrained into the alloy as a bifilm, first attracts the oxy-sulfide nuclei to nucleate on it. Then, these nuclei serve as the underlay for the nucleation of flake and nodular graphite. This theory of graphite nucleation is also mentioned in the works of I. Riposan et al. [11]. F. Hsu et al. [12] and R. Dojka et al. [13] noted the importance of a gating system for manufacturing good-quality castings according to John Campbell’s bifilm theory. The conducted research on ductile irons revealed that turbulent filling from the top results in oxide bifilms entrained in ductile iron castings. F. Hsu et al. [12] concluded that floating bifilms are the sources of intrinsic cracks in castings. Ductile iron castings may be weakened by bifilm inclusions, resulting in their premature fracture. These results show that turbulent filling significantly influences the elongation of ductile iron. The authors’ recent work presented in the paper confirms the results of cast iron investigations in the area of bifilm defects. In the paper, the crystallization of alloyed ductile iron (without the addition of magnesium) with oxide bifilm inclusions is discussed. The phenomenon of simple graphite and bifilm segregation towards the heat center of the castings was also documented.

## 2. Materials and Methods

Tests were carried out based on a two-stage metallurgical process of a liquid metal. Experimental melting was carried out in a medium-frequency induction furnace (PI25, ELKON Sp. z o.o., Rybnik, Poland) with a capacity of 25 kg. Steel scrap with a low sulfur content was used as a charge. Table 1 presents the results of the charge material chemical content analysis. Steel scrap metal was used as a charge material for the test.

The remaining components added during melting included: Ferrosilicon FeSi75 and synthetic graphite with a carbon content above 99.35%. The steel scrap was prepared appropriately for each melting. Before the weighing process, the steel scrap was cleaned of oxides and other impurities. Then, it was dried at 250 °C for 2 h inside a resistance furnace (DW40, Mario Di Maio SpA, 11-20122 Milano, Italy). For each melt, the measured portion of ferrosilicon FeSi75 was annealed at 650 °C for 2 h in a resistance chamber furnace (Curing Furnace F-120, Mario Di Maio SpA, 11-20122 Milano, Italy). Another stage included initial melting, which consisted of a melt of steel scrap with added graphite carburizer and ferrosilicon. After removing the slag, the prepared and melted material was poured into a steel casting mold. In the next step, the material prepared this way was used in the proper melt. Double melting was conducted to eliminate gas dissolved in the liquid alloy, which is recommended in the literature [14,15]. During the initial melting, samples for carbon content analysis were collected. The planned carbon content was 0.5%. The second stage of melting consisted of melting a previously prepared charge in an induction furnace and correcting the carbon content. During the melting of the charge, the metal bath was degassed [14]. This method was based on overheating liquid metal to 1400 °C, followed by a gradual reduction in the temperature in the furnace to approx. 1200 °C to remove gases from the bath, which is described in [16]. After the liquid alloy reached the temperature of 1200 °C, it was heated to approx. 1350 °C. Then, the liquid metal was poured into a ladle, the bottom of which was covered with FeTi67 foundry alloy to degas the metal bath.

The chemical content analysis of samples was done in several stages, and the results are presented in Table 2. During the first stage, an analysis of the chemical composition was performed using a Leco GDS 500 spectrometer (Model No 607-500, Leco Corporation, 3000 Lakeview Ave, St. Joseph, MI, USA). Due to the significant quantity of alloying additives, which in this case was silicon, the estimated Si content exceeded the allowable limits for the measured content of this element and for the used standard (maximum Si level circa 5%). Taking the above into account, it was decided to further analyze the Si content using the gravimetric method. Additional analysis for carbon and sulfur content was also conducted using a CS125 carbon-sulfur analyzer (Leco Corporation, 3000 Lakeview Ave, St. Joseph, MI, USA). The results of the aforementioned analyses are presented in Table 2.

The samples for the metallographic tests were cast from the analyzed melt. These samples were cast in molds made of resin-covered sand, whose shape corresponds to the standardized samples for testing impact strength.

Metallographic tests were performed using scanning electron microscopy (Phenom Pro-X with EDS system–Phenom-World B.V. Dillenburgstraat 9T Eindhoven, 5652 AM, The Netherlands). Cross-sections of the analyzed samples were used for testing.

## 3. Results

### 3.1. SEM Analysis

**Figure 2:**
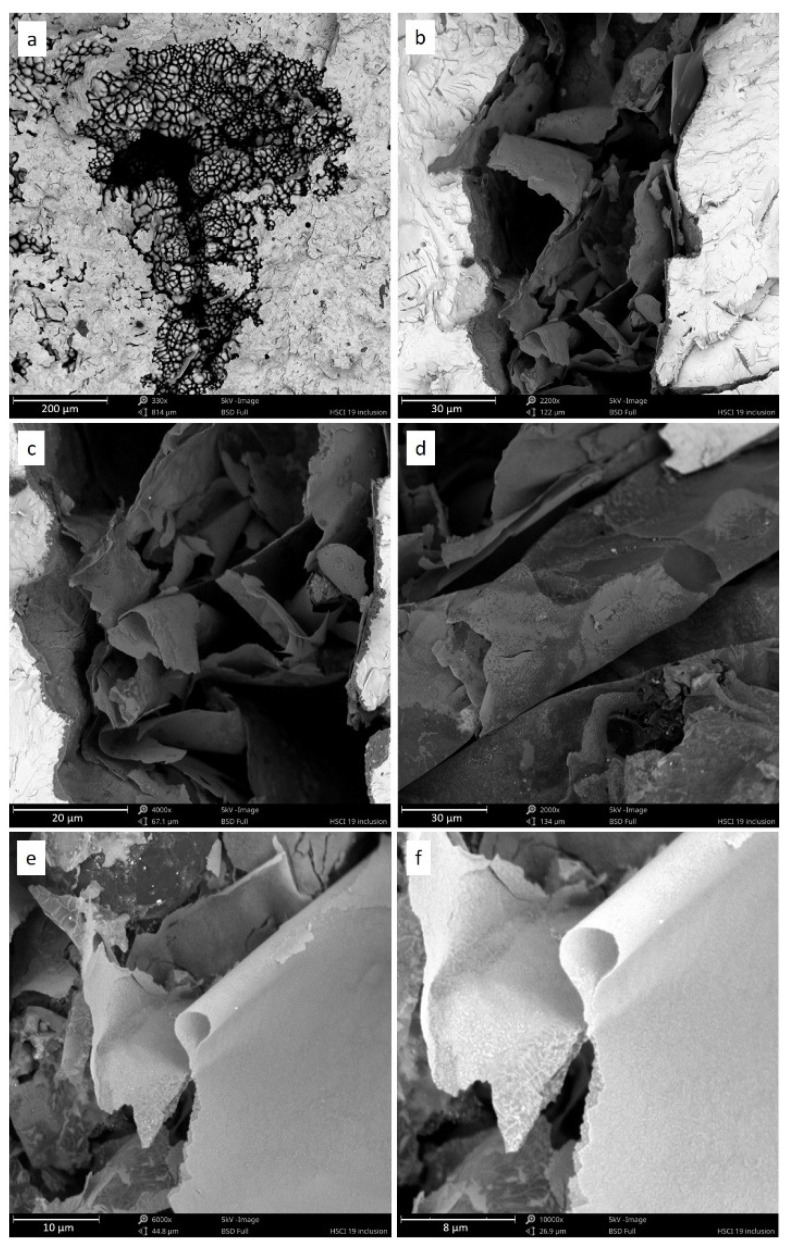
Contraction cavity (**a**) with a visible dendritic structure. Contraction cavities (**b**–**d**) filled with bifilm-type oxide inclusions. A single bifilm rolled into a tube (**e**,**f**).

Figure 2a presents the contraction cavity cross-section, where the dendritic macrostructure of silicon ferrite is visible. Figure 2b–d presents the cross-section of the contraction cavity with disorganized bifilm inclusions inside. Inclusions of this type tend to increase the degree of expansion. Figure 2e,f shows that a non-expanded bifilm formed a distinct complex shape.

**Figure 3:**
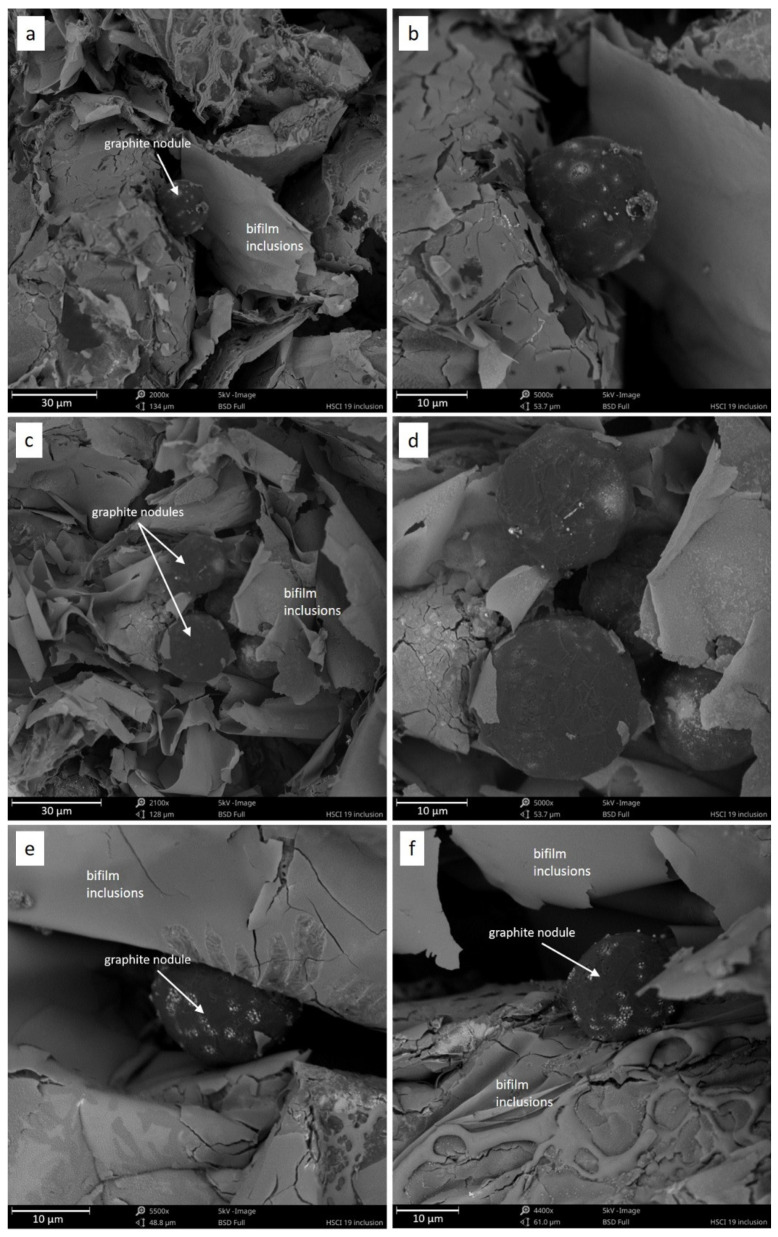
Contraction cavities (**a**–**f**) filled with bifilms inclusions and nodule graphite precipitates.

An interesting phenomenon was captured in Figure 3, which showed that besides bifilm inclusions, the contraction cavities also contained spheroidal graphite precipitates. The external surface of the graphite precipitates suggests that these are primary precipitates [16,17,18,19]. These precipitates were pushed out of the liquid alloy towards the contraction cavity. Bifilm inclusions that were also floating in the liquid alloy reached the contraction cavity on the crystallization front.

The surface of spheroidal graphite precipitates was smooth. According to previous studies [17,18,20,21,22], such a structure suggests that these are primary graphites that crystallized directly from the liquid. In such a case, the graphite crystallization process was stopped due to the discharge of spheroids from the liquid alloy. That is why the surface of precipitations is smooth. They were not disturbed by the secondary crystallization process. The chemical content of the analyzed alloy is strongly hypereutectic, which supports the hypothesis that this is primary graphite.

### 3.2. EDS Analysis

**Figure 4:**
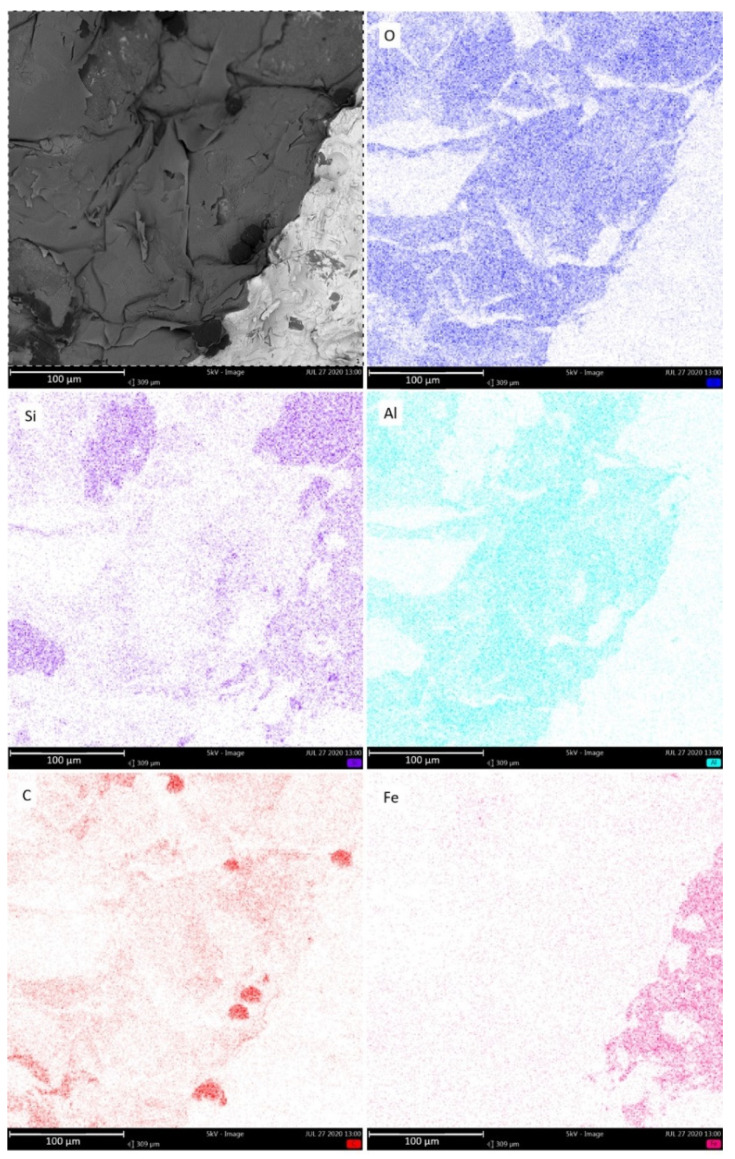
The EDS analysis of the contraction cavity’s surface covered in bifilms with spheroidal graphite extrusions.

The EDS analysis was conducted for a selected cross-section of a fragment with a flat surface. Figure 4 shows an image of the measurement area. The composition of several elements such as oxygen, silicon, aluminum, carbon, and iron was determined. Based on these analyses, it can be concluded that the bifilm layer was primarily rich in oxygen, aluminum, and silicon. Due to possible errors during the analysis, the chemical content of this layer cannot be unambiguously specified. The preparation of metallographic micro-sections was hindered in this case. The spheroidal graphite precipitates may have been lost during polishing, and the oxide inclusions (bifilms) may have been damaged as well.

**Figure 5:**
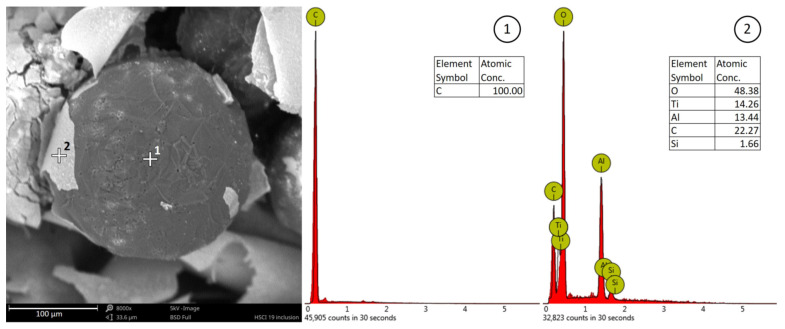
Chemical content local analysis of the dark extrusion (1)–graphite, and the light extrusion (2)–bifilm.

Figure 5(1) shows the results of a local spectral analysis of the spheroidal graphite precipitates. The result explicitly confirms that the precipitation consists primarily of carbon. The oxide inclusion was also analyzed, as shown in Figure 5(2). The results of the oxide film analysis did not provide a clear assessment of the chemical composition, but the layer was rich in oxygen, aluminum, silicon, carbon, and titanium.

## 4. Discussion

In addition to clusters, the liquid cast iron submicroscopic graphite particles were formed from the graphite precipitates in the metallic charge material. The graphite structure type caused the individual flat layers of carbon atoms to retain their bonds at temperatures as high as 2000 °C, while the bonds between the layers were easily broken. The destruction of graphite crystals during melting and overheating of the alloy began with a loss of bonds between the packets of flat layers. These packets then dissociated into individual flat layers, which was caused by the iron, oxygen, and hydrogen ions entering them. Depending on the charge material, overheating temperature, etc., the liquid cast iron consisted of blocks of graphite packets, packets of flat layers or areas covered with melted packets in the form of individual thin graphite molecules forming ionic bonds between carbon atoms and solvent atoms, as well as other elements and individual carbon atoms. The aforementioned graphite formations maintained an unstable equilibrium in a liquid alloy. The temperature and time that the alloy can withstand are decisive factors for the size of homonymous groupings and atoms.

As the liquid alloy cooled down to the liquidus temperature, the flat layers began compounding into packets, which then compounded into blocks that eventually combined with each other.

In addition to homogeneous germs, heterogeneous germs in the form of various non-metallic inclusions can also play an important role in the graphitization process. These types of germs are most frequently attributed to oxide inclusions, especially SiO_2_ compounds [20,23]. The literature shows oxides identified in the interior of spheroidal graphite, including magnesium, silicon, aluminum, and titanium oxides [20,23]. John Campbell [10] wrote about the role of oxide films (silica bifilms) in the growth of various forms of graphite. The presence of oxides (MgO, SiO_2_, Fe_2_O_3_, MnO) or silicates (Mg_2_SiO_4_, Fe_2_SiO_4_) in liquid ductile iron or the slag bound within has been documented [10,23]. Complex compounds, such as (Mg_2_SiO_4_, Fe_2_SiO_4_) have been recognized in cast iron [24]. Campbell proposed that these oxides are not taut marbles, cubes, rods, etc., but rather films or bifilms with a lower Stokes velocity, which lets them remain in the alloy suspension for a long time. Other researchers came to similar conclusions [25,26]. When there is no Mg, the oxysulfide particles nucleate on oxide bifilms rich in silica, and then graphite nucleates and grows on oxysulfide inclusions, forming flake graphite. The magnesium additives eliminate bifilms rich in silica, and then graphite spheroids grow on oxysulfides, as it is assumed that graphite naturally grows spherically.

**Figure 6:**
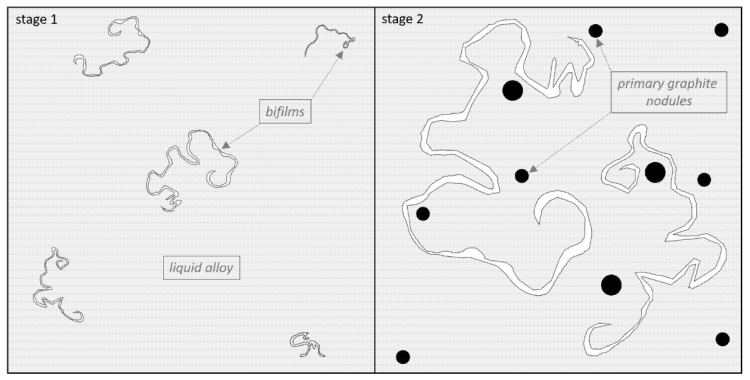
Bifilms in the liquid alloy (stage 1). Graphite crystallization directly from the liquid (stage 2).

Non-metallic inclusions, along with a set of exogenous graphite particles, heavily influence the cast iron’s ability to nucleate individual components of the microstructure, mainly graphite. Graphite spheroids start forming via regular nucleation through very heavy supercooling or irregular nucleation, within inclusions [23]. We noted an interesting phenomenon associated with the formation of an oxide film or bifilm and graphite primary crystallization. The results are in close agreement with theories presented by other researchers mentioned in the paper. According to the conducted studies presented in Figure 2, Figure 3, Figure 4 and Figure 5 and recent works [8], the following mechanism was proposed for the observed phenomena. The scheme in Figure 6 shows that in stage 1 after pouring, only oxide bifilm inclusions are present in the liquid alloy. Then, in stage 2, graphite crystallization occurs, and primary graphite nodules form in the liquid alloy, accompanied by inclusions. Spheroidal graphite is the main phase in the investigated alloy.

According to the theories of D.M. Stefanescu and J. Campbell [10,23], graphite precipitates crystallize on the substrates. Graphite growth in a liquid solution requires not only the intensive diffusion of carbon atoms towards the growing crystal but also the intensive movement of iron atoms in the opposite direction. The graphite eutectic system in spheroidal cast iron crystallizes outside the area of simultaneous growth [27]. Graphite and austenite crystallize separately under significant supercooling conditions. During the first stage, graphite spheroids grow in direct contact with the liquid. However, the eutectic austenite growth does not occur simultaneously. Both phases grow separately, and graphite spheroids grown via austenite dendrites are eventually surrounded by austenite and separated from the metallic fluid [17]. A similar cast iron crystallization pattern was presented by M. Zhu et al., but without the participation of bifilms [27].

**Figure 7:**
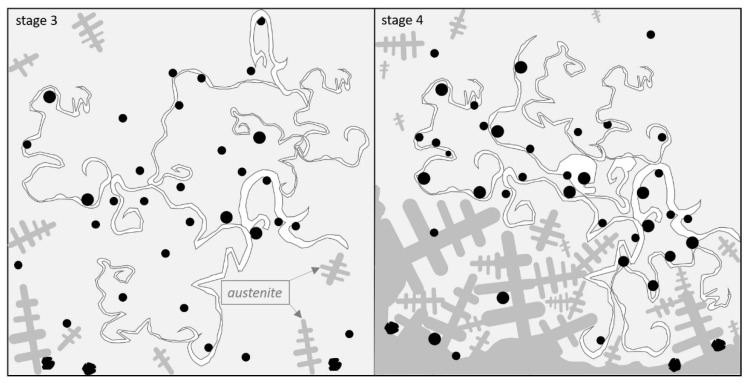
Graphite eutectic system growth in the bifilm environment step by step (stage 3 and satge 4).

Based on reported theories and results [17,27], we can predict the presence of austenite in stage 3 (Figure 7).

Due to a low carbon solubility in the liquid alloy (with a high Si content), the graphite precipitates may be transported on the crystallization front. In addition to graphite nodules, an oxide layer and light bifilms were also pushed by the growing crystals, which led to stage 4 (Figure 7). In this stage, graphite precipitates might be covered and mixed into the double oxide film. The spheroidal graphite nucleation on the surface of the oxide film may be the reason for the accumulation of graphite nodules in the contraction cavity.

If we look again at the oxide inclusions in the companion of graphite presented in Figure 3, we can observe the fragile and torn character of the oxide film. The same torn layer of the oxide film is visible in Figure 5, which may be the result of mixing and turbulence during filling the mold. It may be that when the bifilms seized and mixed with the graphite precipitates, and graphite nodules tore apart the fragile film.

**Figure 8:**
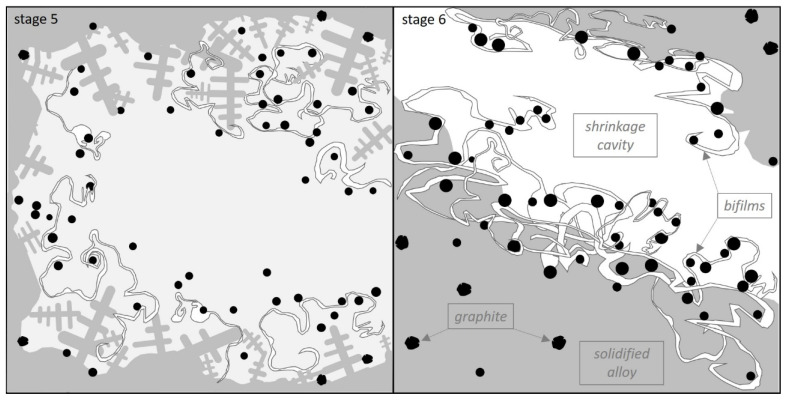
Creation process of shrinkage cavity (stage 5). Shrinkage cavity with primary graphite nodules mixed and trapped with the bifilm inclusions (stage 6).

Moreover, the film may also be damaged by graphite growth on its surface. In stage 5 (Figure 8) it can be noticed, how graphite nodules mixed with the bifilms are pushed on the growing dendrites. Due to the presented crystallization mechanisms, a portion of the precipitated graphite nodules is forced out towards the contraction cavities which is presented in stage 6 (Figure 8).

## 5. Conclusions

This work provided the following observations and conclusions:

- Spheroidal precipitates crystallized directly from the liquid and may nucleate on the substrates created by the bifilms. This hypothesis was indirectly supported by a large number of spheroidal graphite precipitates in the presence of bifilms.
- Spheroidal graphite precipitates were forced out of the liquid. In the discussed case, the precipitates were forced in the direction of the contraction cavities.
- Contraction cavities with no visible bifilm inclusions, as well as spheroidal graphite precipitates, were observed in the sample.
- The smooth surface of spheroidal graphite precipitates suggests that primary graphite crystallized directly from the liquid.
- Spheroidal graphite precipitates occurring during the mixing in the liquid alloy ripped the bifilms.
- Some bifilm precipitates occurring in the alloy did not have the time to develop and formed tube-like shapes.
- Primary graphite was forced out of the metallic fluid. There are oxide inclusions in the way of the graphite spheroids. Spheroid graphite precipitates were captured in oxide lattices. Both were discharged from the liquid alloy towards the contraction cavity.

## Figures and Tables

**Table 1:** Chemical analysis of steel scrap used in the research.

Chemical Composition, % of Weight						
C 0.080	Cr 0.037	Si 0.001	Mn 0.594	Ni 0.024	Mo 0.01	S 0.006
Co 0.010	Cu 0.004	Al 0.046	Sb 0.009	As 0.108	B 0.00	P 0.016
Pb 0.002	Nb 0.031	Sn 0.009	Ti 0.001	W 0.011	V 0.006	Fe bal 98.995

**Table 2:** Chemical composition of high-silicon cast iron.

Chemical Composition, % of Weight											
	**^(1)^ Si**	**^(2)^ C**	**^(2)^ S**	**P**	**Mn**	**Mo**	**Cu**	**Mg**	**Ti**	**Fe _bal_**	**^(3)^ C_e_**
HSCI	18.70	0.52	0.003	0.022	0.301	0.022	0.064	0.00	0.027	80.34	6.32

^(1)^ Si analysis by weight method, ^(2)^ carbon and sulfur analysis by CS 125 Leco, and ^(3)^ eutectic carbon equivalent calculated for C, P, and Si.

## Data Availability

Data sharing is not applicable to this article.
